# Supplementary material for: Multi‐dataset identification of innovative feature genes and molecular mechanisms in keratoconus
Source: J Cell Mol Med. 2024 Sep 19;28(18):e70079. doi: 10.1111/jcmm.70079 (PMC11412914; doi:10.1111/jcmm.70079)
Supplement: Supplementary file 2 — Material S2. [file JCMM-28-e70079-s002.zip › 7.GSEA analysis/1.ARL11/KEGG_BETA_ALANINE_METABOLISM.html]

Details for gene set KEGG\_BETA\_ALANINE\_METABOLISM[GSEA]

|  || Dataset | ARL11.group.cls#h\_versus\_l |
| Phenotype | group.cls#h\_versus\_l |
| Upregulated in class | h |
| GeneSet | KEGG\_BETA\_ALANINE\_METABOLISM |
| Enrichment Score (ES) | 0.34159473 |
| Normalized Enrichment Score (NES) | 0.95504344 |
| Nominal p-value | 0.50576925 |
| FDR q-value | 1.0 |
| FWER p-Value | 1.0 |
Table: GSEA Results Summary

  

Fig 1: Enrichment plot: KEGG\_BETA\_ALANINE\_METABOLISM      
 Profile of the Running ES Score & Positions of GeneSet Members on the Rank Ordered List

  

| SYMBOL | TITLE | RANK IN GENE LIST | RANK METRIC SCORE | RUNNING ES | CORE ENRICHMENT || 1 | AOC2 | na | 1449 | 0.342 | 0.0926 | Yes |
| 2 | GAD1 | na | 1955 | 0.300 | 0.1958 | Yes |
| 3 | DPYD | na | 3337 | 0.225 | 0.2445 | Yes |
| 4 | CNDP1 | na | 4027 | 0.200 | 0.3032 | Yes |
| 5 | DPYS | na | 4971 | 0.167 | 0.3416 | Yes |
| 6 | ALDH7A1 | na | 10913 | 0.048 | 0.1894 | No |
| 7 | HIBCH | na | 11013 | 0.047 | 0.2052 | No |
| 8 | ACADM | na | 11858 | 0.038 | 0.1957 | No |
| 9 | ALDH3A2 | na | 13283 | 0.025 | 0.1643 | No |
| 10 | GAD2 | na | 15039 | 0.011 | 0.1180 | No |
| 11 | MLYCD | na | 16660 | -0.001 | 0.0717 | No |
| 12 | ABAT | na | 17429 | -0.006 | 0.0521 | No |
| 13 | ECHS1 | na | 17627 | -0.008 | 0.0494 | No |
| 14 | HADHA | na | 19491 | -0.020 | 0.0038 | No |
| 15 | SRM | na | 20072 | -0.024 | -0.0035 | No |
| 16 | SMS | na | 21570 | -0.036 | -0.0325 | No |
| 17 | EHHADH | na | 24876 | -0.074 | -0.0985 | No |
| 18 | ALDH1B1 | na | 25126 | -0.078 | -0.0752 | No |
| 19 | ALDH9A1 | na | 25157 | -0.078 | -0.0453 | No |
| 20 | ALDH2 | na | 31150 | -0.226 | -0.1292 | No |
| 21 | UPB1 | na | 32517 | -0.288 | -0.0552 | No |
| 22 | AOC3 | na | 32726 | -0.302 | 0.0577 | No |
Table: GSEA details [plain text format]

  

Fig 2: KEGG\_BETA\_ALANINE\_METABOLISM      
 Blue-Pink O' Gram in the Space of the Analyzed GeneSet

  

Fig 3: KEGG\_BETA\_ALANINE\_METABOLISM: Random ES distribution      
 Gene set null distribution of ES for **KEGG\_BETA\_ALANINE\_METABOLISM**
